# Rapid multiple protein sequence search by parallel and heterogeneous computation

**DOI:** 10.1093/bioinformatics/btae151

**Published:** 2024-03-28

**Authors:** Jiefu Li, Ziyuan Wang, Xuwei Fan, Ruijie Yao, Guoqing Zhang, Rui Fan, Zefeng Wang

**Affiliations:** CAS Key Laboratory of Computational Biology, Shanghai Institute of Nutrition and Health, University of Chinese Academy of Sciences, Chinese Academy of Sciences, 320 Yueyang Road, Shanghai 200031, China; School of Information Science and Technology, ShanghaiTech University, Shanghai 201210, China; School of Information Science and Technology, ShanghaiTech University, Shanghai 201210, China; Institute of Intelligent Computing Technology, Chinese Academy of Sciences, 88 Jinjihu Avenue, Suzhou, Jiangsu 215000, China; CAS Key Laboratory of Computational Biology, Shanghai Institute of Nutrition and Health, University of Chinese Academy of Sciences, Chinese Academy of Sciences, 320 Yueyang Road, Shanghai 200031, China; Bio-Med Big Data Center, Shanghai Institute of Nutrition and Health, University of Chinese Academy of Sciences, Chinese Academy of Sciences, 320 Yueyang Road, Shanghai 200031, China; School of Information Science and Technology, ShanghaiTech University, Shanghai 201210, China; CAS Key Laboratory of Computational Biology, Shanghai Institute of Nutrition and Health, University of Chinese Academy of Sciences, Chinese Academy of Sciences, 320 Yueyang Road, Shanghai 200031, China; Bio-Med Big Data Center, Shanghai Institute of Nutrition and Health, University of Chinese Academy of Sciences, Chinese Academy of Sciences, 320 Yueyang Road, Shanghai 200031, China; School of Life Science, Southern University of Science and Technology, Shenzhen, Guangdong 518055, China

## Abstract

**Motivation:**

Protein sequence database search and multiple sequence alignment generation is a fundamental task in many bioinformatics analyses. As the data volume of sequences continues to grow rapidly, there is an increasing need for efficient and scalable multiple sequence query algorithms for super-large databases without expensive time and computational costs.

**Results:**

We introduce Chorus, a novel protein sequence query system that leverages parallel model and heterogeneous computation architecture to enable users to query thousands of protein sequences concurrently against large protein databases on a desktop workstation. Chorus achieves over 100× speedup over BLASTP without sacrificing sensitivity. We demonstrate the utility of Chorus through a case study of analyzing a ∼1.5-TB large-scale metagenomic datasets for novel CRISPR-Cas protein discovery within 30 min.

**Availability and implementation:**

Chorus is open-source and its code repository is available at https://github.com/Bio-Acc/Chorus.

## 1 Introduction

The most commonly used protein homology search tool is BLASTP from the BLAST suite (Basic Local Alignment Search Tool) (Altschul *et al.* 1990) as evidenced by over 100 000 citations. This highlights the broad applicability and importance of sequence homolog detection and multiple sequence alignment (MSA) in various fields of biological research. The differences between protein sequences, which are quantified by MSA, serve as a fundamental basis for downstream analyses, including the annotation of unknown genes, the inference of evolutionary relationships, and the discovery of novel proteins.

Although protein sequence query is widely used and has a large user base, the current main service options are limited to either using the free service provided by NCBI or deploying on the user's own server. In addition, current protein query algorithms, such as MMseqs2 (Steinegger and Söding 2017), DIAMOND (Buchfink *et al.* 2014, 2021), and HHblits (Remmert *et al.* 2012), have been designed for CPU architectures, and the limited number of GPU-accelerated BLAST versions, such as H-BLAST (Ye *et al.* 2017), perform less optimally than CPU methods. Consequently, as the size of the database to be searched continues to grow rapidly and users require personalized searches, this critical task in bioinformatics analysis has been unable to fully leverage the recent rapid advancements in heterogeneous computing, especially in GPU over the past few years. Both DIAMOND and MMseq2 can be embedded in a large-scale cloud computing solution (Ma *et al.* 2018, Mirdita *et al.* 2022), however, this has raised high learning and usage cost and huge computational resource consumption for users.

In this study, we have developed a highly parallel MSA algorithm, Chorus. Chorus can leverage the full power of heterogeneous computing architectures and GPUs, achieving over 100× speedup compared to BLASTP without sacrificing sensitivity. Chorus is fully open source, supporting multiple output formats that can be easily integrated into existing analysis processes. With Chorus, sequence analysis that previously required a large server or supercomputing system can now be completed in minutes on an average desktop workstation equipped with a GPU.

## 2 Materials and methods

The first step of many mainstream query methods is to index the reference sequence database. However, while various sophisticated indexing techniques can reduce the size of the reference collection, as the content of the database continues to evolve and personalized data query needs arise, relying on large amounts of computational resources to repeatedly build indexes is no longer a good option.

**Figure 1.**
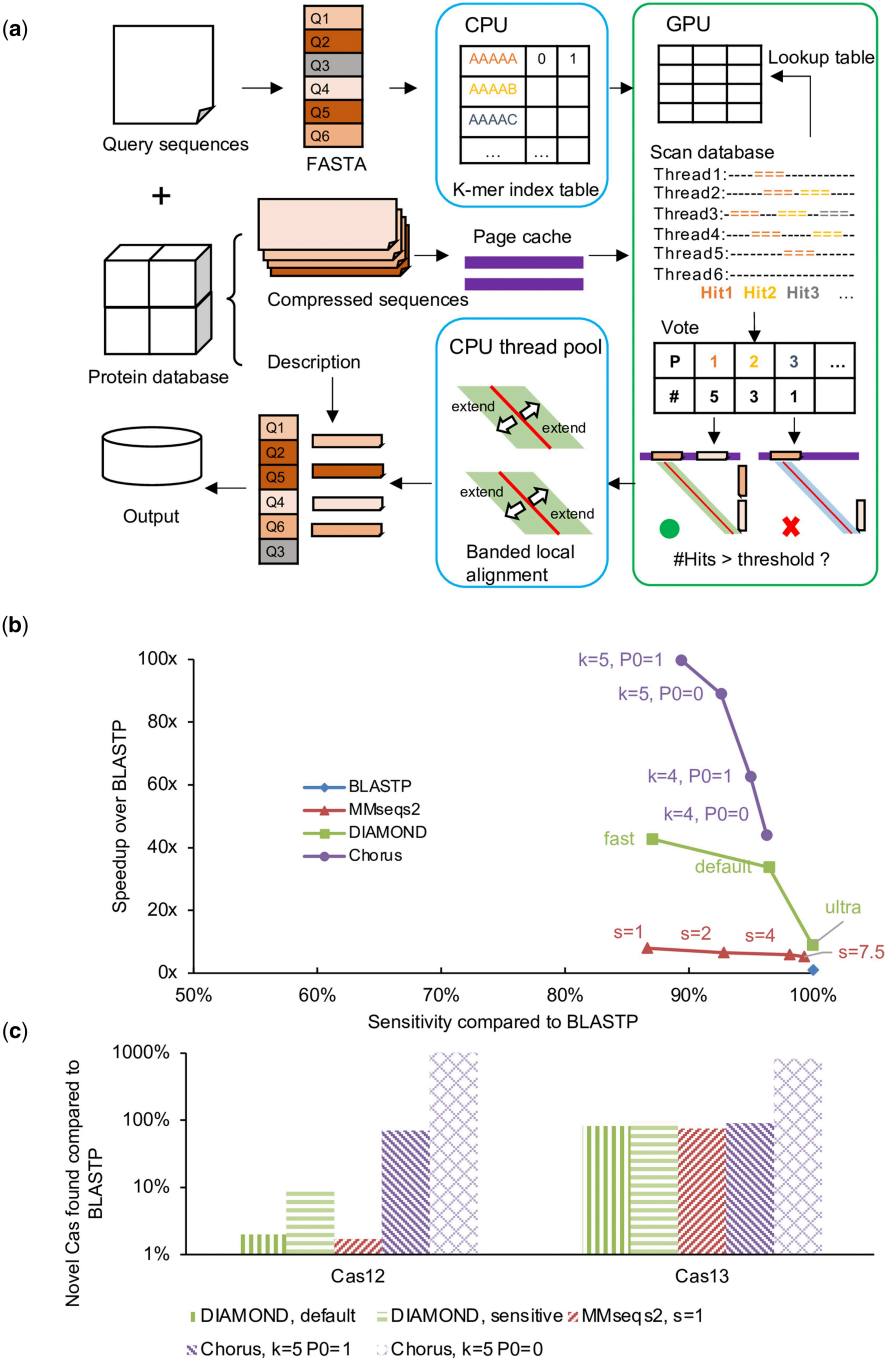
Overview of Chorus Overview of Chorus. (a) First, Chorus creates a k-mer index table for the input protein query sequences, and copy to the GPU together with the preprocessed protein sequences database. GPU threads parallel scan the database and look-up the index table, recording the alignment position where the hit occurs. When the number of hits in a alignment position is greater than the threshold, then pass this HSP candidate back to the host memory. The CPU performs parallel banded local alignment on this HSP by using a thread pool, and finally outputs the alignment result. (b) Sensitivity and speed-up of the proposed method relative to BLASTP, using a randomly selected subset of 100 sequences (1000–1200 aa) from Swiss-Prot against the NCBI NR database (226 GB, December 2021). Sensitivity is measured as the coverage of alignments over half the query length, averaged across all queries. (c) Novel Cas12/13 proteins identified in comparison with BLASTP. The search utilized an 87 GB database with 228 554 945 sequences derived from metagenome-assembled genomes (MAGs). Queries comprised 193 known Cas12 and 109 Cas13 sequences

To address these issues, Chorus only builds a k-mer table for query sequences, ensuring that the table size remains relatively constant, even as the number of sequences increases. The reference sequence database is split into several sub-databases that can be queried by batches. For each batch, the sub-database is small enough to store in device memory. We designed a ‘seed-and-vote’ algorithm to ensure that each batch only traverses the sub-database only once, reducing the number of computational expensive massive I/O operations required for querying the reference sequence database (Fig. 1a). To reduces false-positive bias, Chorus adopted a tantan masking method (Frith 2011) to process database if needed. Furthermore, to mitigate the latency resulting from data communication between the CPU and GPU, we have developed a pipelined task scheduling model. This model is designed to ensure that various subtasks can optimally utilize the available computational resources concurrently. By adopting this approach, we can significantly reduce the time required for data transfers between the CPU and GPU, resulting in a more efficient and streamlined workflow (Supplementary Fig. S1a).

To fully leverage the capabilities of heterogeneous computing systems, we designed a strategy that take the full advantage of the strengths for both CPUs and GPUs. We allocate computationally intensive and logically complex tasks, such as preprocessing large query and database sequences and local alignment (Supplementary Fig. S1b, d, and e), to CPUs. Meanwhile, tasks that demand concurrent operations on massive data, notably global seeding on the database, voting for candidate sequences and filtering (Supplementary Fig. S1c, Supplementary Algorithms S1–S3), are executed on GPUs or GPU-like devices. This approach allows us to efficiently leverage the strengths of CPU and GPU and maximize the overall system performance and resource usage.

## 3 Results

### 3.1 Computational resource dependency and occupancy

Firstly, we evaluated the performance of Chorus on different hardware configurations. (Supplementary Fig. S4) When the data exceeded >1000 sequences, we found that Chorus performed better on the consumer GPU RTX3090 than A6000. The higher memory frequency of the RTX3090 allowed for more efficient data throughput, which compensated for its slower processing speed of single batches of data. We further examined the system resource usage of the program at different data volumes and observed that local alignment, which is mainly handled by the CPU, only became a performance bottleneck when the input data contained several thousand items. (Supplementary Fig. S5) This problem is expected to be improved in the near future as hardware technology continues to advance.

### 3.2 Performance comparison with competing methods

Next, we evaluated the performance of Chorus for large-scale protein sequences matching by comparing it to other existing tools on publicly available datasets. We systematically analyzed the impact of different sequence lengths and quantities on the performance of all methods. We selected BLASTP (Altschul *et al.* 1990) as the benchmark for testing, H-BLAST (Ye *et al.* 2017) as a representative of the currently available GPU version of the BLAST tool and included MMseqs2 (Steinegger and Söding 2017), DIAMOND (Buchfink *et al.* 2014, 2021). For testing, we used NCBI's NR database (226GB, as of December 2021) and randomly selected multiple groups of proteins with a total length of 9000 amino acids from the Swiss-Port database. The average length of each group ranged from 100 to 600. We used all 56 threads by default for all CPU-based methods (Table 1).

**Table 1.** Competing method.

Method	CPU/GPU	Ref.	Exact software version
Chorus	CPU-GPU	This study	
MMseqs2	CPU	Steinegger and Söding (2017)	45111b641859ed0ddd875b94d6fd1aef1a675b7e
DIAMOND	CPU	Buchfink *et al.* (2021)	2.0.14
H-blast	CPU-GPU	Ye *et al.* (2017)	v1.1
BLASTP	CPU	Altschul *et al.* (1990)	2.12.0+

As shown in Supplementary Fig. S6, Chorus and DIAMOND demonstrated improved speedup compared to BLASTP. Specifically, Chorus achieved the best acceleration, with an average speed up of around 10 times compared to BLASTP across different input sizes. Notably, Chorus was almost unaffected by changes in input data size. However, MMseqs2 failed to achieve the desired speedup due to the small number of sequences in the dataset. While H-BLAST can maintain the same output as BLAST, it failed to outperform the 56-threaded BLAST in terms of performance. Moreover, its frequent runtime errors (Segmentation Fault) during querying of large-scale protein databases were excluded from subsequent analysis.

The choice of query sequences and databases can significantly affect performance and results. (Supplementary Fig. S7) To assess the performance and search sensitivity of Chorus with competing methods, we conducted similar evaluations using test data from MMseqs2 and DIAMOND. For MMseqs2 test, the experiments employed a set of 6370 sequences from the SCOP (Structural Classification of Proteins) database (version 1.75), ranging from amino acids 26 to 18 095, with a total length of 2.56 million amino acids. To minimize experimental complexity, the authors of the MMseqs2 used these sequences as queries against the smaller UniRef50 database (June 2015 version, size 14 GB). The DIAMONDS authors randomly selected sequences of varying length (tens to thousands of amino acids) from the SCOPe (Structural Classification of Proteins extended) database, using the smaller annotated version of the UniRef50 database (September 2019, size 3.4 GB) (Table 2).

**Table 2.** Benchmark datasets.

Query	Database	Ref.
Swissport various Length	NCBI-NR (12/2021 226G)	Ye *et al.* (2017)
SCOP 6370	UniRef50 (06/2015)	Steinegger and Söding (2017)
SCOP e-annotated	UniRef50	Buchfink *et al.* (2021)
MMseq2 benchmark	MMseq2 benchmark	Steinegger and Söding (2017)
Sampled from UniRef50 (June 2015 version)	UniRef90 Mar 2023 version	

As the number of query sequences increases, the relative performance of each tool compared to BLAST improves. Notably, even though both DIAMOND and MMseqs2 utilize 56 threads for acceleration, they perform similarly or even worse than BLASTP when there are fewer sequences. However, the DIAMOND-fast run faster than Chorus when query exceeds 1000. This is consistent with hitting the performance bottleneck for hardware reason mentioned above. Chorus consistently outperforms all other methods before the cross-over points and achieves a speedup of approximately 600 times when querying thousands of sequences in both the MMseqs2 and DIAMOND datasets (Supplementary Fig. S7).

To further study the cross-over point, we built a new benchmark dataset. The database is UniRef90 (Mar 2023 version, size 75G). The sequences in the query set were randomly extracted from UniRef50 (June 2015 version), encompassing a size of 14 GB, with each sequence having a length of 1000 amino acids. Supplementary Figure S8 illustrates the outcomes, indicating that when the cumulative length of sequences surpasses 250 000, with a total of 250 sequences each spanning 1000 amino acids, DIAMOND exhibits superior performance in comparison to Chorus. Interestingly, MMseq2 exhibited greater time consumption than initially anticipated. These findings showed that, for scenarios requiring the processing of exceptionally large volumes of data, DIAMOND may be the better alternative, particularly when hardware resources are constrained.

### 3.3 Trade-off between speed and sensitivity

In this evaluation, the true positive (TP) can be clearly defined (often the output by BLASTP), and the definition of true negative (TN) is more difficult. Therefore, most tools have chosen sensitivity as an indicator to evaluate the results. We conducted a similar analysis to that of DIAMOND's authors to assess the trade-off between performance and sensitivity among all the competing methods. As illustrated in Fig. 1b, Chorus demonstrated the highest acceleration over BLASTP. Even in its slowest mode (*k* = 4, P0 = 1), Chorus provided a superior acceleration effect (43-fold faster than BLASTP) than DIAMOND (42-fold faster than BLASTP). Since Chorus employs a completely different sequence similarity comparison strategy than MMseqs2 and DIAMOND, the output scores characterizing sequence similarity by Chorus cannot be directly compared with this two software and can only be used for similarity ranking within the Chorus output. Nonetheless, the sensitivity levels remain relatively consistent across all methods.

We utilized the MMseqs2 benchmark to assess the reliability of homology search results, employing the ROC5 sensitivity metric. This metric evaluates the methods' ability to generate a correctly ranked list of homologous hits on the precision–recall curve. As shown in Supplementary Fig. S9a, Chorus outperforms other methods in terms of ROC5 values. Furthermore, Supplementary Fig. S9b indicates that Chorus achieves a slightly higher count of high-scoring false positives. However, as shown in Supplementary Fig. S9c, Chorus identifies the most results, inevitably leading to an increased number of false positive results. Nevertheless, it maintains sensitivity and performance within an acceptable range. These findings collectively demonstrate that Chorus achieves accelerated performance without compromising accuracy and sensitivity, while simultaneously reducing the number of false positive identifications.

### 3.4 Case study: novel Cas12/13 discovery

We evaluated the effectiveness of our method by applying it to the computational pipeline discovery of a novel CRISPR-Cas protein from large metagenomic datasets (Supplementary Fig. S10a). While many Cas9 proteins have been identified, there are still numerous unexplored Cas12 and Cas13 proteins that have the potential to be discovered by computational pipelines. Our approach utilized a screening process similar to current mainstream methods (Konermann *et al.* 2018, Rybarski *et al.* 2021, Xu *et al.* 2021, Yang *et al.* 2022). Specifically, we first identified CRISPR arrays, predicted open reading frames (ORFs) near the arrays, and compiled a database of the predicted ORFs. We then used a collection of known Cas12 and Cas13 protein sequences as query sequences to search the database for new candidate gene editing proteins. The query results were subsequently clustered and filtered to identify the most promising candidates. To improve the efficiency of our method, we replaced the homology search tool from BLASTP (Altschul *et al.* 1990) to Chorus, and we also evaluated the effect of replacing BLASTP with MMseqs2 (Steinegger and Söding 2017) and DIAMOND (Buchfink *et al.* 2014, 2021). Our database was composed of ∼1.5 TB of data, which included NCBI genome (∼975 GB), SGB (∼163 GB), Tara Ocean (∼22 GB), and GMGC (∼305 GB). Traditional BLASTP-based methods would have taken several days to complete the analysis, but our approach significantly reduced the computation time.

To compare the performance of different query tools, we measured the time consumption of each method. As shown in Supplementary Fig. S10b, Chorus outperformed the other methods, completing the homology search in only 33 min. DIAMOND's default mode came in second, taking more time than Chorus but still much less than the total running time of over 85 h for BLASTP. MMseqs2 took the longest time of all methods evaluated. We then tested the consistency of the tools in different protein identification processes and compared their results to the BLASTP benchmark. (Supplementary Fig. S10c) Our analysis indicated that Chorus, DIAMOND, and MMseqs2 had better consistency with BLASTP for the identification of Cas12 and Cas13 proteins.

We conducted a further comparison of the number of novel proteins identified by each method relative to BLASTP (Fig. 1c). Chorus exhibited the highest sensitivity, enabling the discovery of the most proteins in a reasonable amount of time, particularly for identifying novel Cas12 or Cas13 proteins. Additionally, the majority of known proteins are identified using BLASTP, and tools that produce results somewhat different from BLASTP are more likely to discover previously unknown proteins. This observation underscores the importance of integrating multiple approaches in mining new proteins using computational biology methods. Utilizing a variety of tools helps reduce biases introduced by individual methods, resulting in more comprehensive and reliable results in novel protein discovery.

Overall, our results indicate that Chorus is an effective and efficient protein MSA tool that can be applied to large-scale functional annotation, such as novel CRISPR-Cas12/13 protein identification.

## 4 Discussion and conclusion

Here, we present a novel protein multiple sequence query system Chorus. It is designed to address the challenge of rapidly growing sequence data volume by leveraging a parallel model and heterogeneous architecture. With Chorus, users can concurrently query thousands of protein sequences against TB-level protein databases on a desktop workstation without incurring expensive time and computational costs. Our software is best suited for searching thousands of protein sequences simultaneously, each with an average length of thousands of amino acids. This task is not affected by the size of the database, making it suitable for most academic and industrial needs. Furthermore, the analysis can be performed on a personal desktop workstation equipped with a consumer GPU, eliminating the need for a high-performance server typically required for such analyses.

Nevertheless, the program’s running time is influenced by various factors, including the hardware environment, computational tasks, and the dataset being processed. Conducting a systematic evaluation is challenging due to these multiple influences. We intentionally selected a diverse range of data types to encompass various scenarios and conducted a comprehensive evaluation using readily available equipment. However, it's important to note that there are inevitably numerous scenarios that were not included in our testing. In certain computing systems equipped with high-end CPUs, our acceleration may not be as effective as some of the excellent CPU-only methods, particularly when the amount of data increases.

Currently, one of the main performance bottlenecks in Chorus is the local alignment module processed in CPU. We plan to develop a GPU-specific banded local alignment module and address these limitations through future optimizations. Additionally, we aim to develop multiple sequence matching tools that support nucleic acid sequences, creating a complete multiple sequence toolkit built on a heterogeneous computing architecture.

## Supplementary Material

btae151_Supplementary_Data

## Data Availability

The data associated with this article are available in its source code repository. The benchmark data are listed in Table 2.
