# Supplementary material for: Rapid multiple protein sequence search by parallel and heterogeneous computation
Source: Bioinformatics. 2024 Mar 28;40(4):btae151. doi: 10.1093/bioinformatics/btae151 (PMC11021808; doi:10.1093/bioinformatics/btae151)
Supplement: btae151_Supplementary_Data [file btae151_supplementary_data.zip › Algorithms.docx]

**Algorithm 1:**

| **Algorithm 1: GPU Seed** |
| --- |
| **INPUT:** Database sequences S, k-mer index table of query sequences T  **OUTPUT:** Voting hash table H  **BEGIN**  Initialize hash table H  For each GPU Threads parallel do:  s_begin, s_end are the start and end positions of sequences that allocated from S  For s_offset←s_begin to s_end do:  Read k-mer that started with aa at S[s_offset] from global memory  Calculate the code of k-mer: X←$\sum_{i=0}^{k-1} X_{i}\ll5i$  Look up index table for this k-mer: Hits←T(X)  If Hits not EMPTY:  For each (q,i) in Hits:  Calculate alignment position: p←len(q)+s_offset-i  **Vote(H[q],p)**  Return H  **END** |

**Algorithm 2:**

| **Algorithm 2：GPU Vote** |
| --- |
| **INPUT:** Voting hash table H, alignment position p  **OUTPUT:** Modified H  **BEGIN**  Aggregate neighbouring hits: p←pack(p)  Calculate Murmur hash value: first_slot←Hash(p) mod size(H)  slot←first_slot  do  slot←slot mod size(H)  Access the hash table slot: prev←atomicCAS(H[slot].key, EMPTY, p)  if (prev==EMPTY or prev==key):  Access success, and increase the number of votes: atomicAdd(H[slot].value)  break  else:  Access the next slot by linear probing method: slot←slot+1  While slot≠first_slot  Return H  **END** |

**Algorithm 3:**

| **Algorithm 3：GPU Filter** |
| --- |
| **INPUT:** Voting hash table H, filter threshold P  **OUTPUT:** HSP list R, number of HSP n  **BEGIN**  Initialize HSP list R, n←0  For each GPU Threads parallel do:  h_begin, h_end is the start and end position of scanned region that allocated from H  For h_offset←h_begin to h_end do:  If H[h_offset].value ≥ P do:  r_offset←atomicAdd(n)  R[r_offset]←H[h_offset].key  Return R, n  **END** |
