# Supplementary material for: Rapid multiple protein sequence search by parallel and heterogeneous computation
Source: Bioinformatics. 2024 Mar 28;40(4):btae151. doi: 10.1093/bioinformatics/btae151 (PMC11021808; doi:10.1093/bioinformatics/btae151)
Supplement: btae151_Supplementary_Data [file btae151_supplementary_data.zip › Supplementary_Rapid multiple protein sequence search by parallel and heterogeneous computation.docx]

# Supplementary materials

**Supplementary figure**

**Supplementary figure S1. Design principle of Chorus.**

**
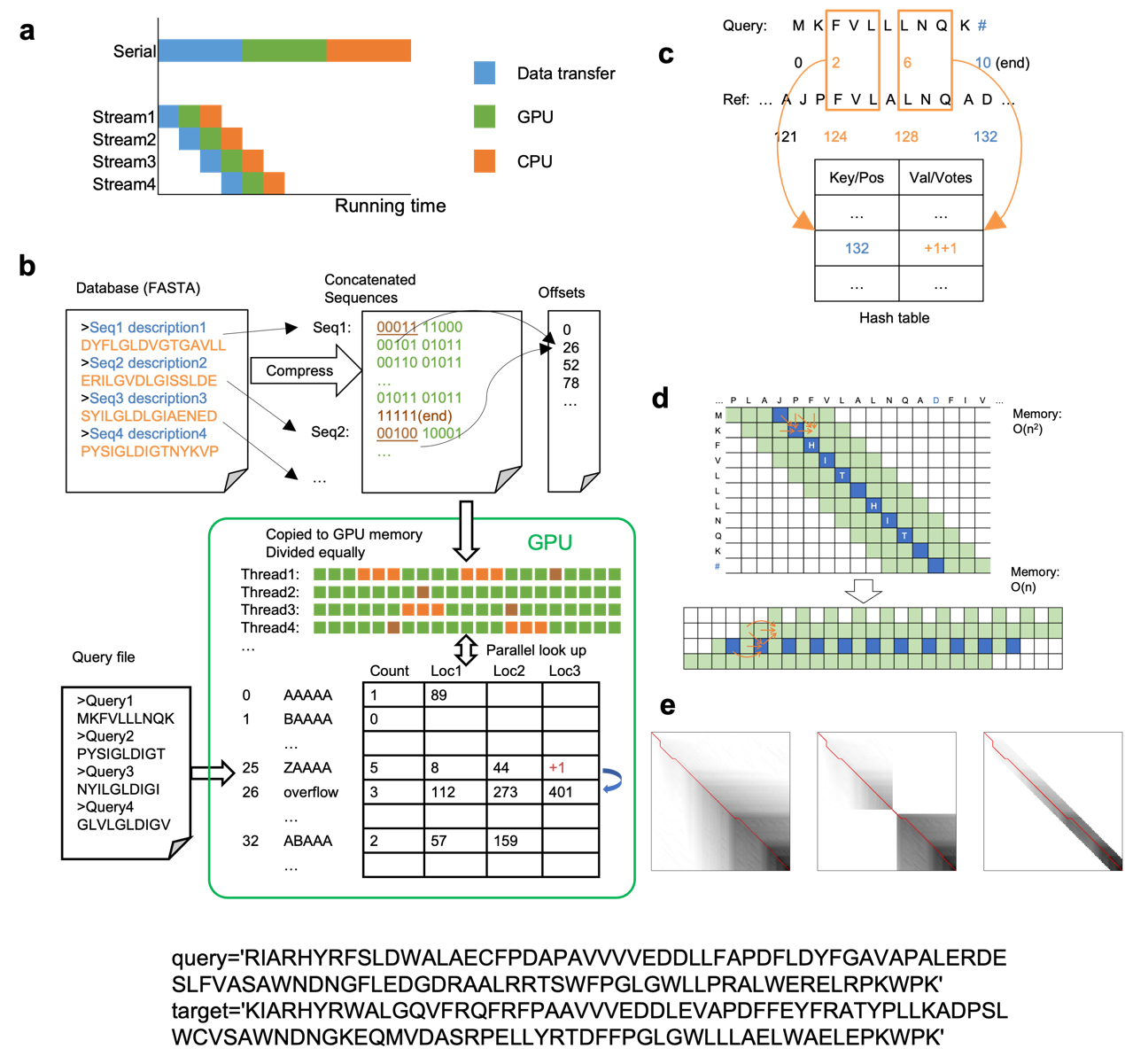
**

1. Running time schema for CPU & GPU collaboration in Chorus. The total running time of a serial workflow is the sum of data transfer, GPU and CPU calculations. By dividing the task into streams, Chorus uses the asynchronous pipelined workflow to maximize the performance of each device.
2. Algorithm schema for GPU parallel seed and vote. Multiple hits that occur at the same alignment position can be parallel recorded at the same slot in the voting hash table.
3. Detailed sequences data processing in Chorus. Sequences in the reference database are compressed and stored contiguously. GPU threads parallel scan the equally divided compressed database sequences and look-up the index table for all k-mers in query sequences.
4. Algorithm schema for banded local alignment. The yellow arrows mark the state dependencies in the Smith-Waterman scoring matrix.
5. Schema of score matrix calculation result. The algorithms are complete Smith-Waterman (left), BLASTP gapped extension (middle) and Chorus banded local alignment (right) respectively. The darker the color, the higher the score, and the red line is the best alignment from the traceback step.

**Supplementary figure S2. Chorus provide output in a variety of formats.**

**
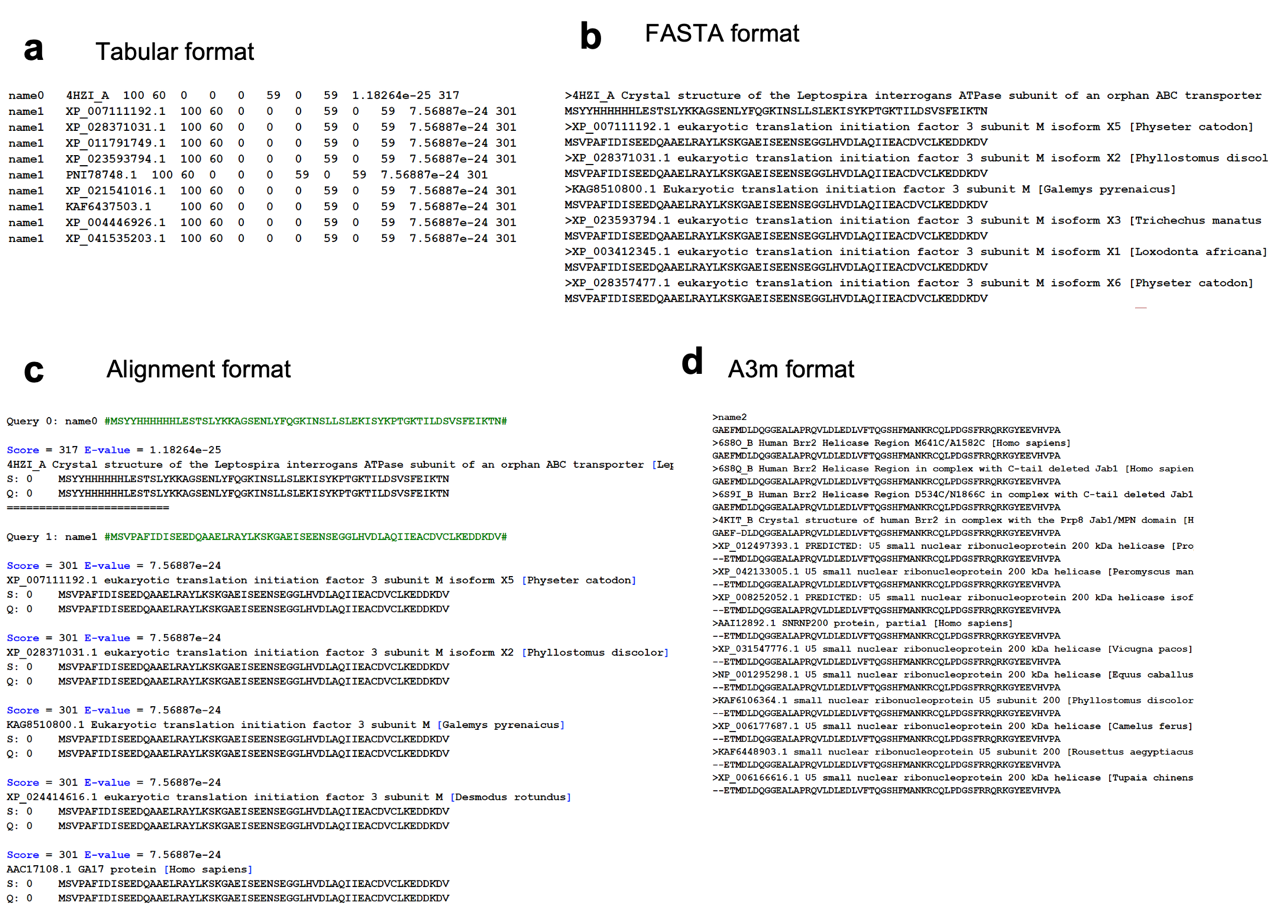
**

1. BLAST standard format.
2. FASTA format for all target sequences.
3. Detailed alignment format.
4. Multiple sequences alignment format a3m.

**Supplementary figure S3. Seed length k and voting threshold P affect the number of HSPs.**

**
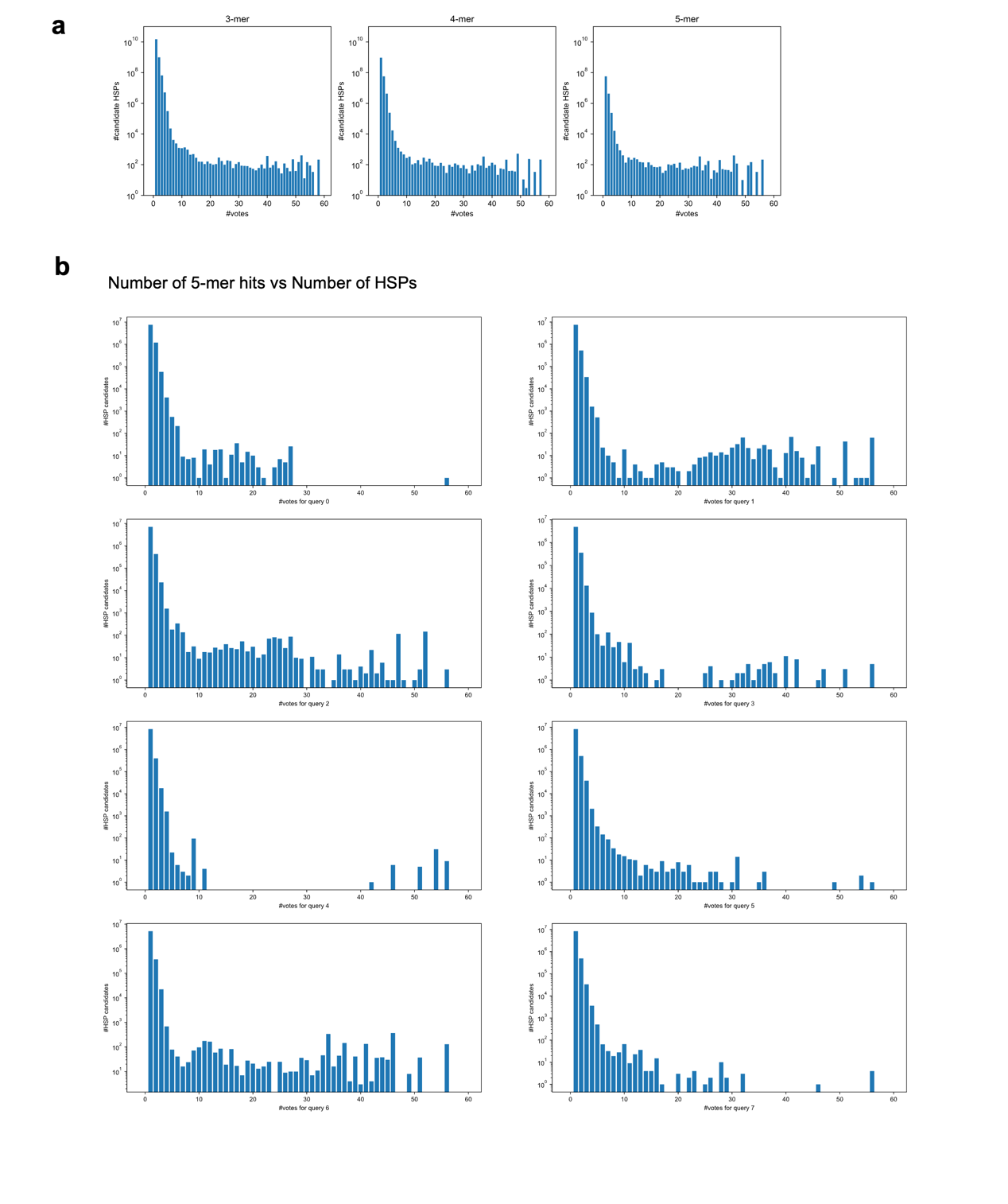
**

**
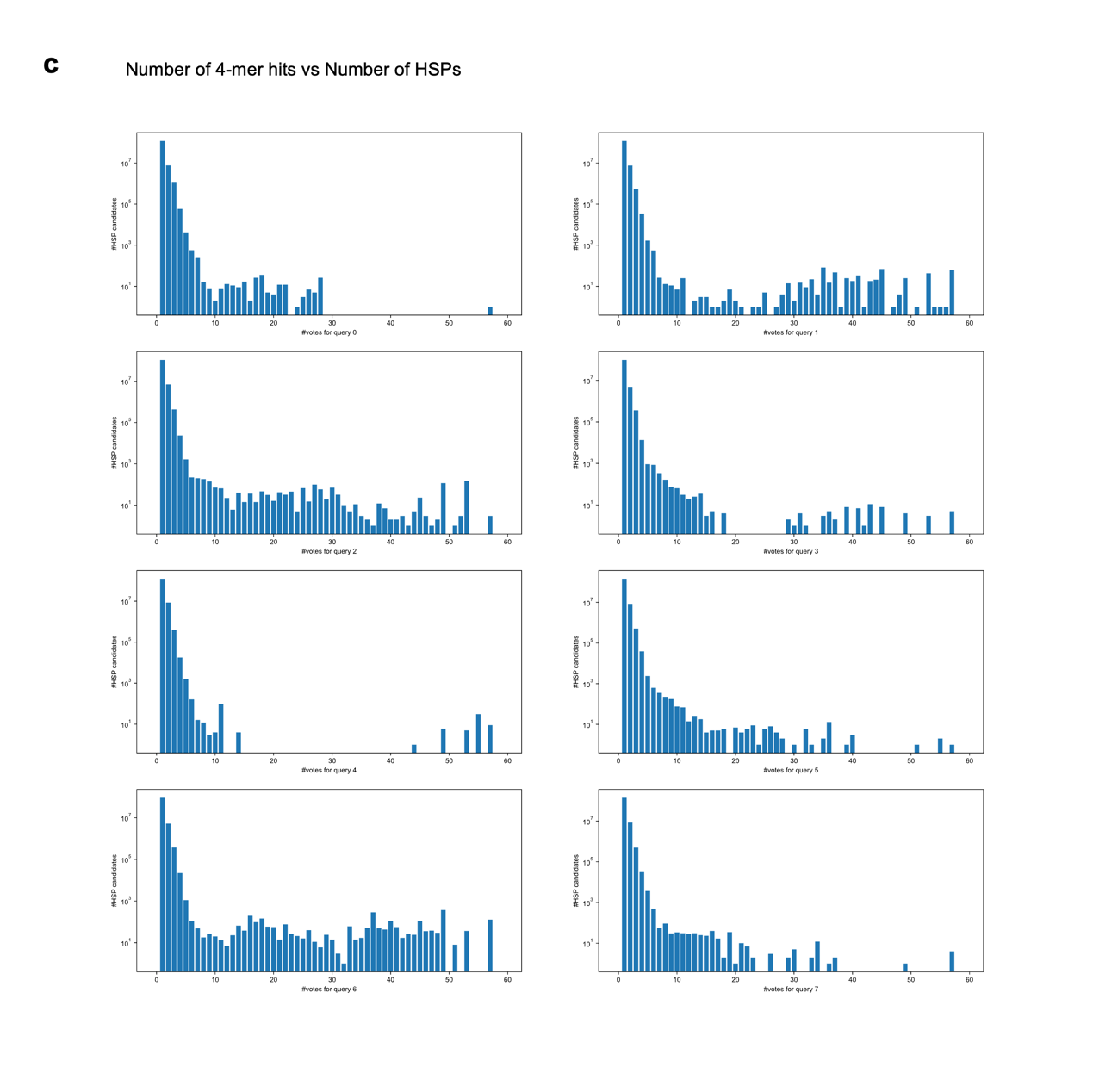
**

**
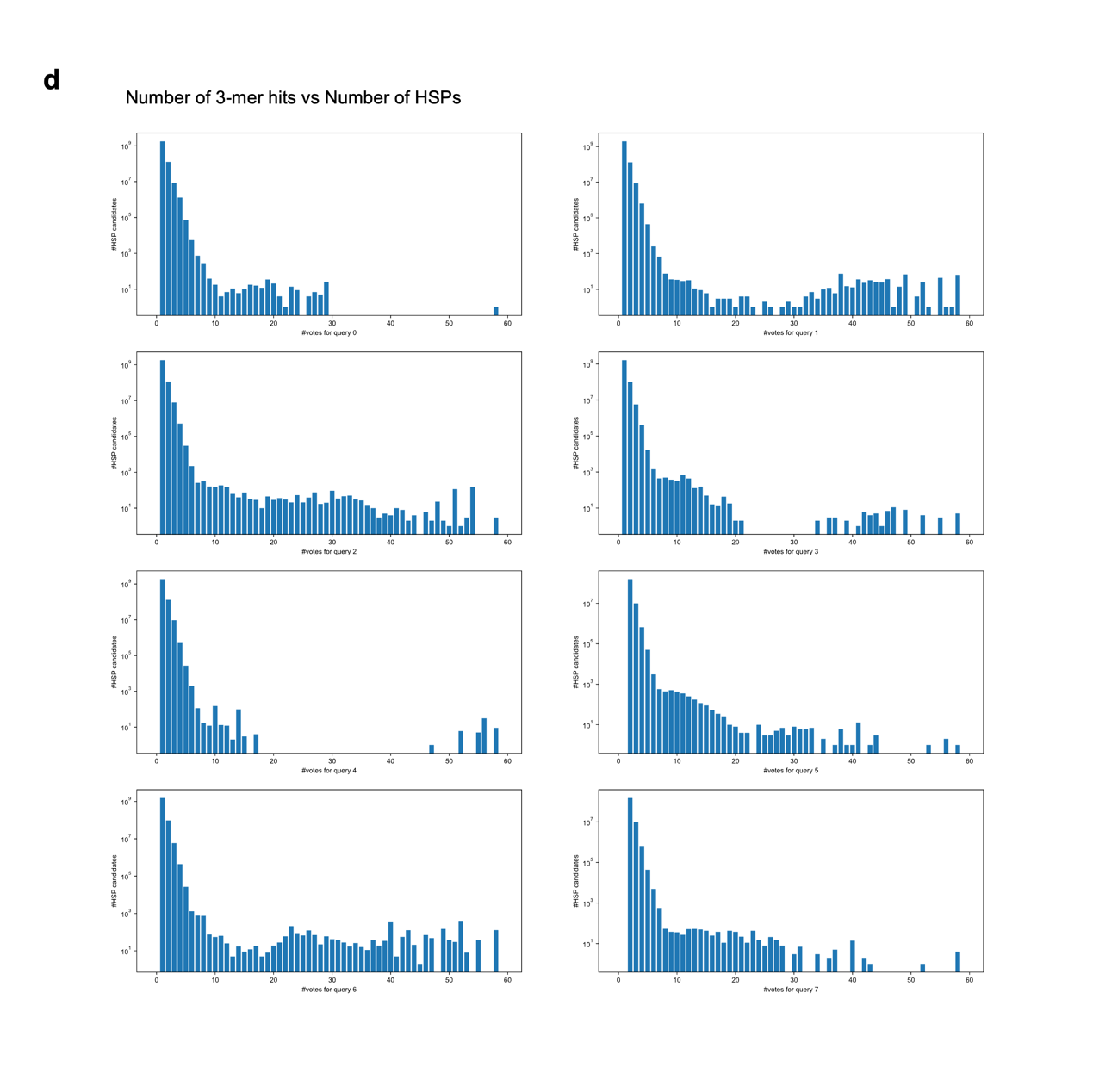
**

**Supplementary figure S4. Performance of Chorus on different devices (GPUs)**, in MMseqs2 benchmark.


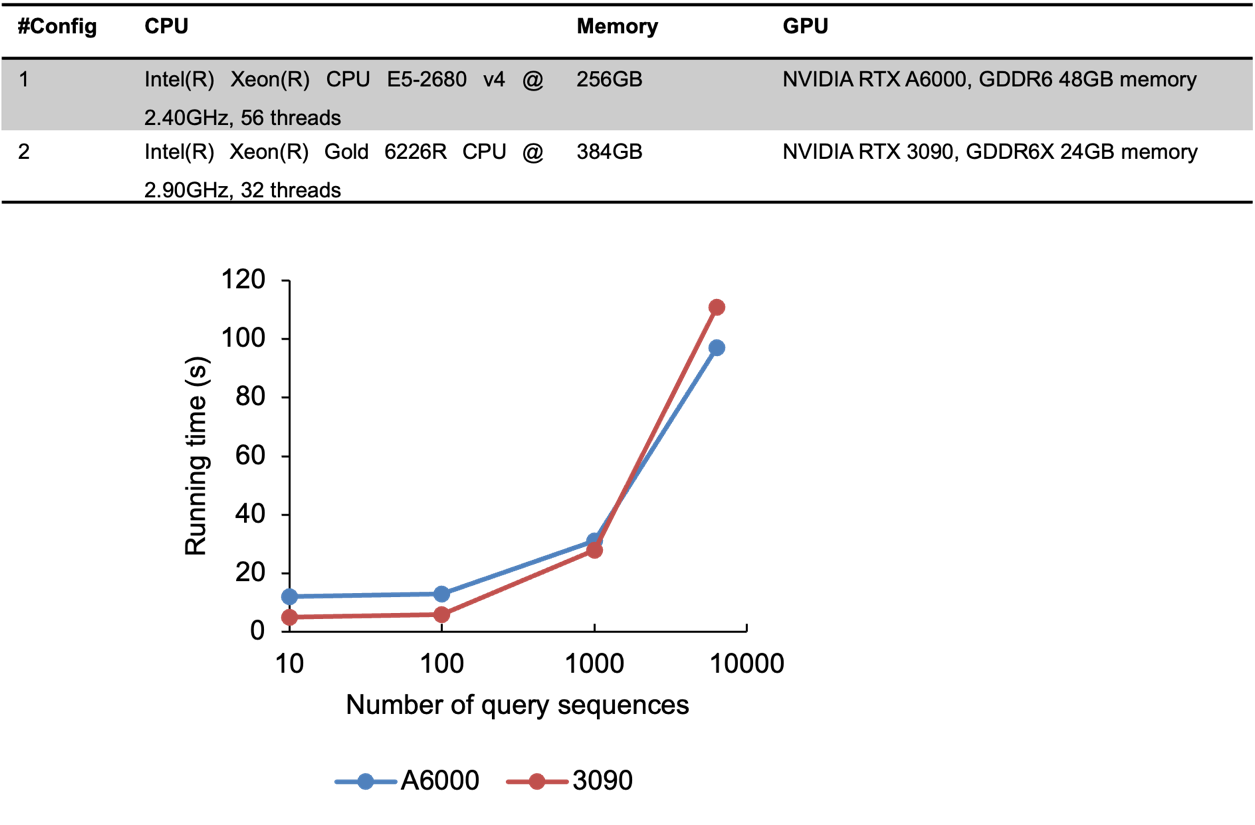


**Supplementary figure S5. Running time profile of Chorus, with different number of query sequences** in DIAMOND benchmark.


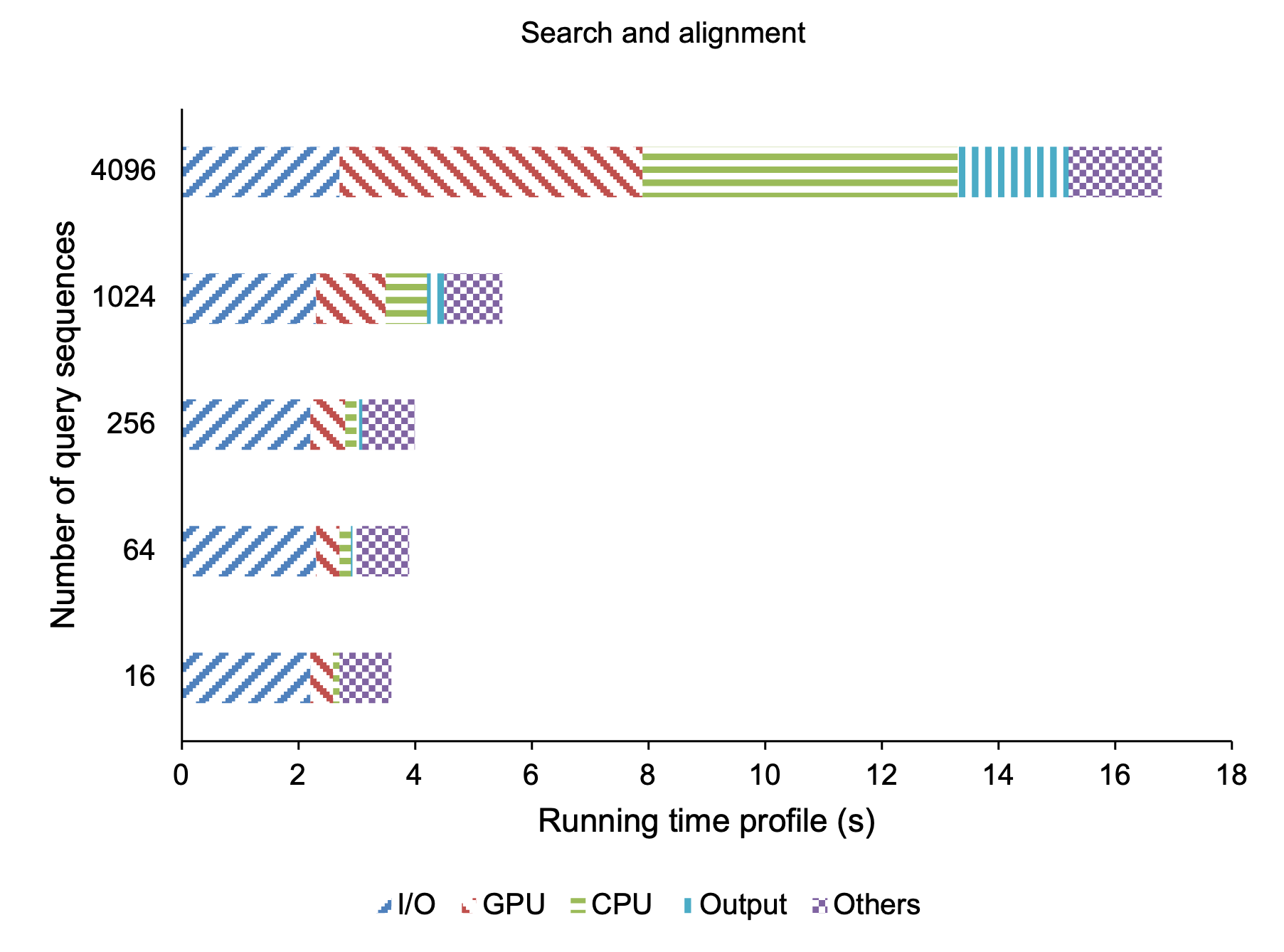


**Supplementary figure S6.** Running time on the H-BLAST benchmark by different methods and sensitive modes. Several protein sequence sets with a total length of 9k are queried in the NCBI nr database. H-BLAST reports runtime errors when querying 1000_9k sequence set.


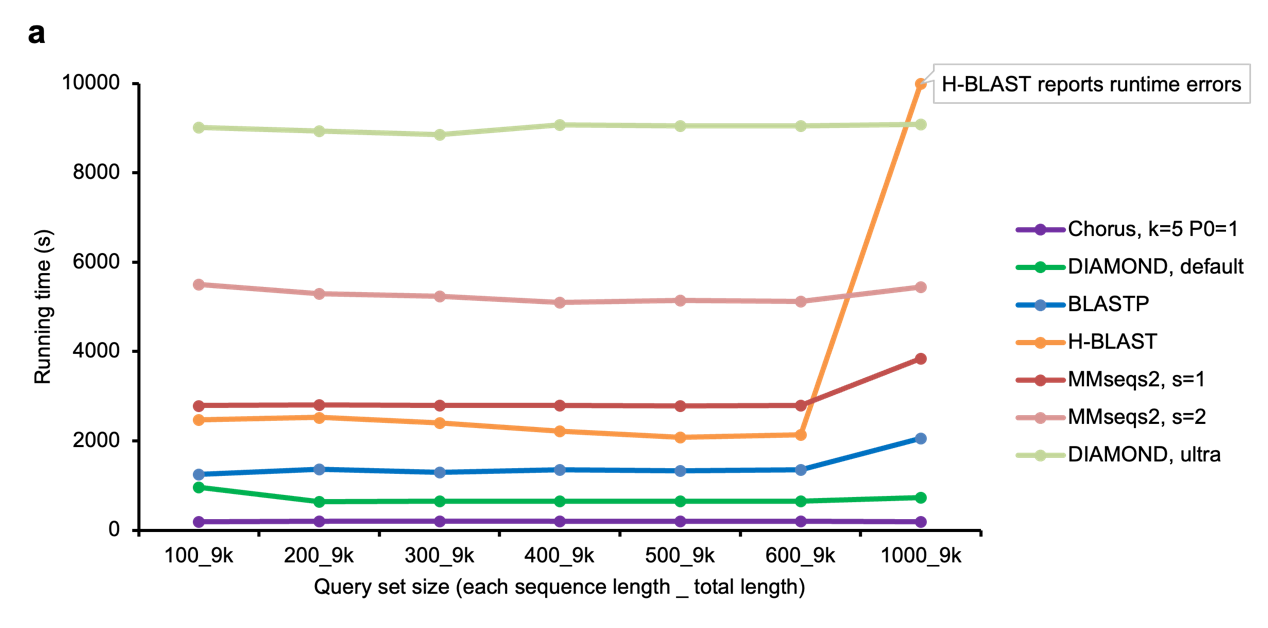


**Supplementary figure S7. Performance and sensitive trade-off with competing methods.**


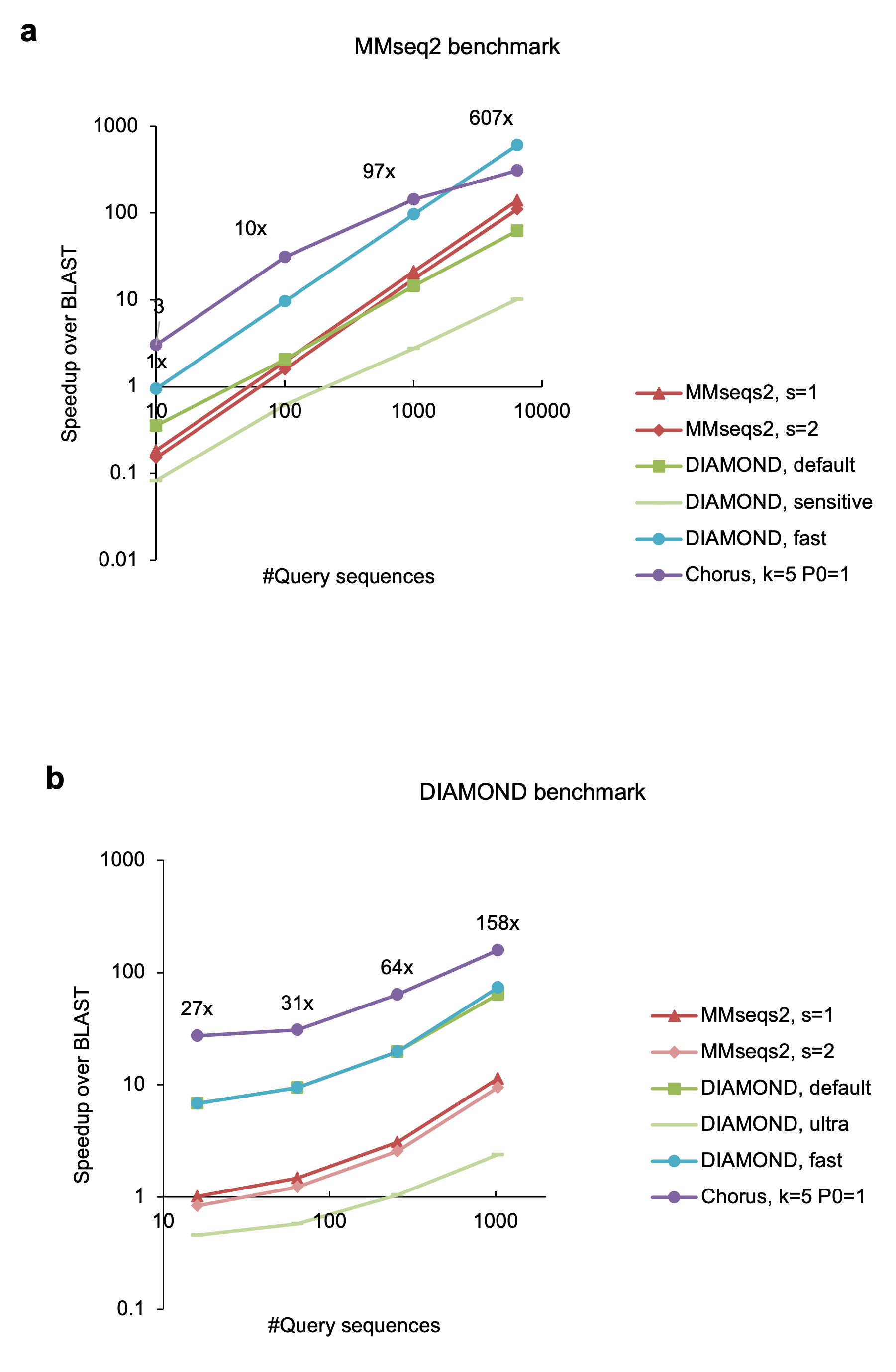


1. Performance speed-up over BLASTP on the MMseqs2 benchmark, with different number of shuffled query sequences. The x,y axes are both logarithmic.
2. Performance speed-up over BLASTP on the DIAMOND benchmark, with different number of shuffled query sequences.

**Supplementary figure S8** **Performance cross-over point with competing methods**


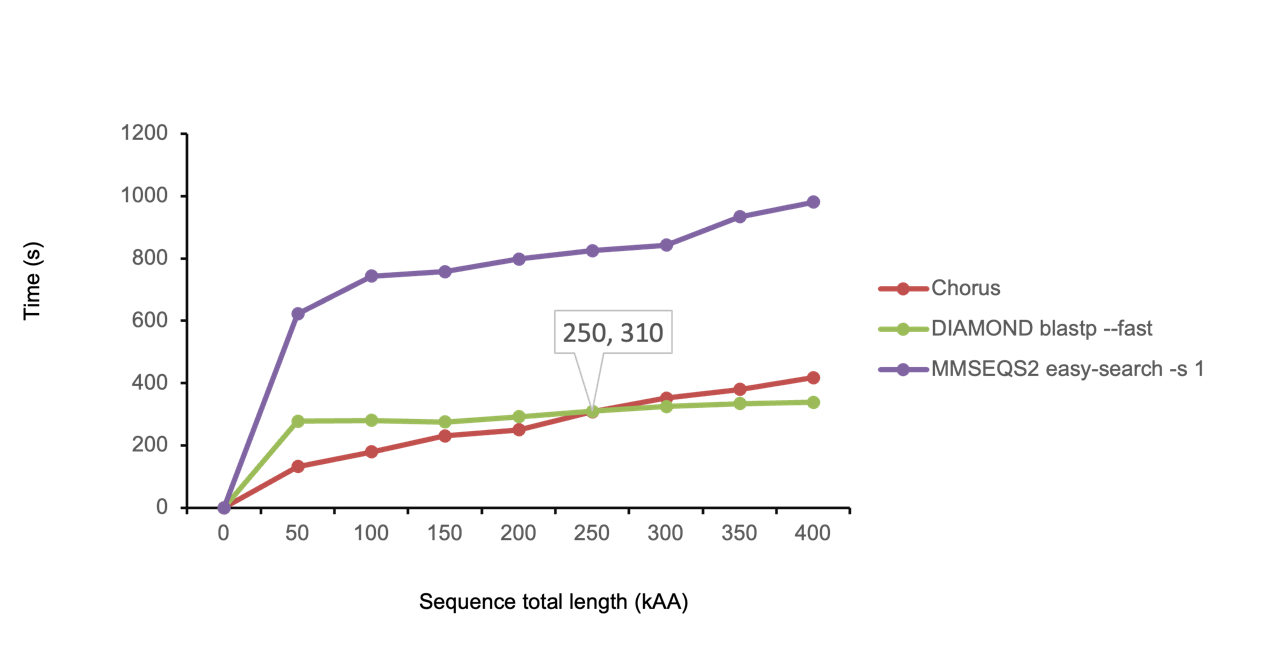


**Supplementary figure S9 Reliability benchmark with competing methods.**


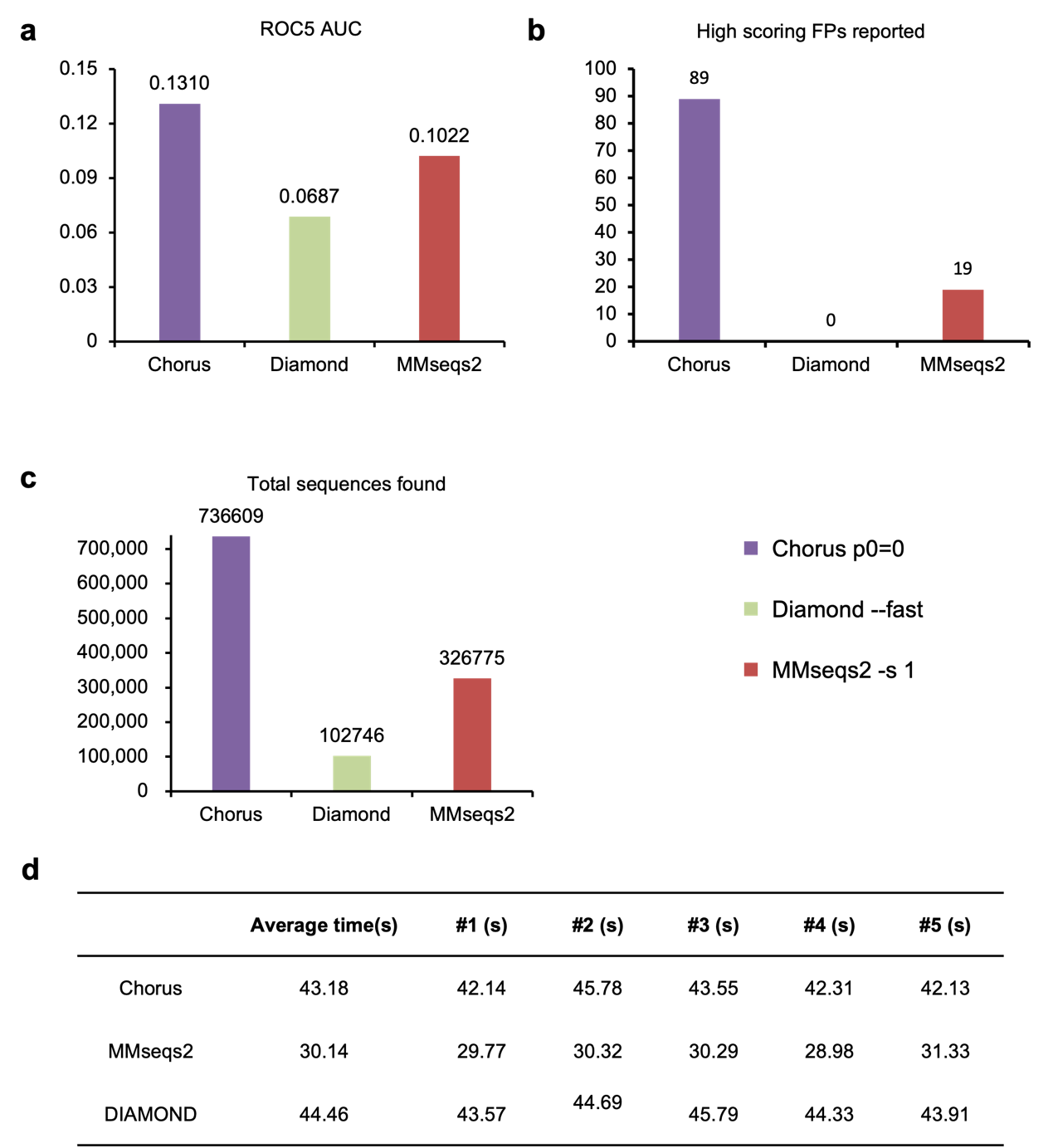


1. ROC5 AUC of MMseq2,DIAMOND and Chorus
2. High scoring false positive (FP)s (e value < 0.001) reported by MMseq2, DIAMOND and Chorus
3. Sequence count of reported by MMseq2, DIAMOND and Chorus
4. The average run-time.

**Supplementary figure S10 Application in novel Cas12/13 discovery**


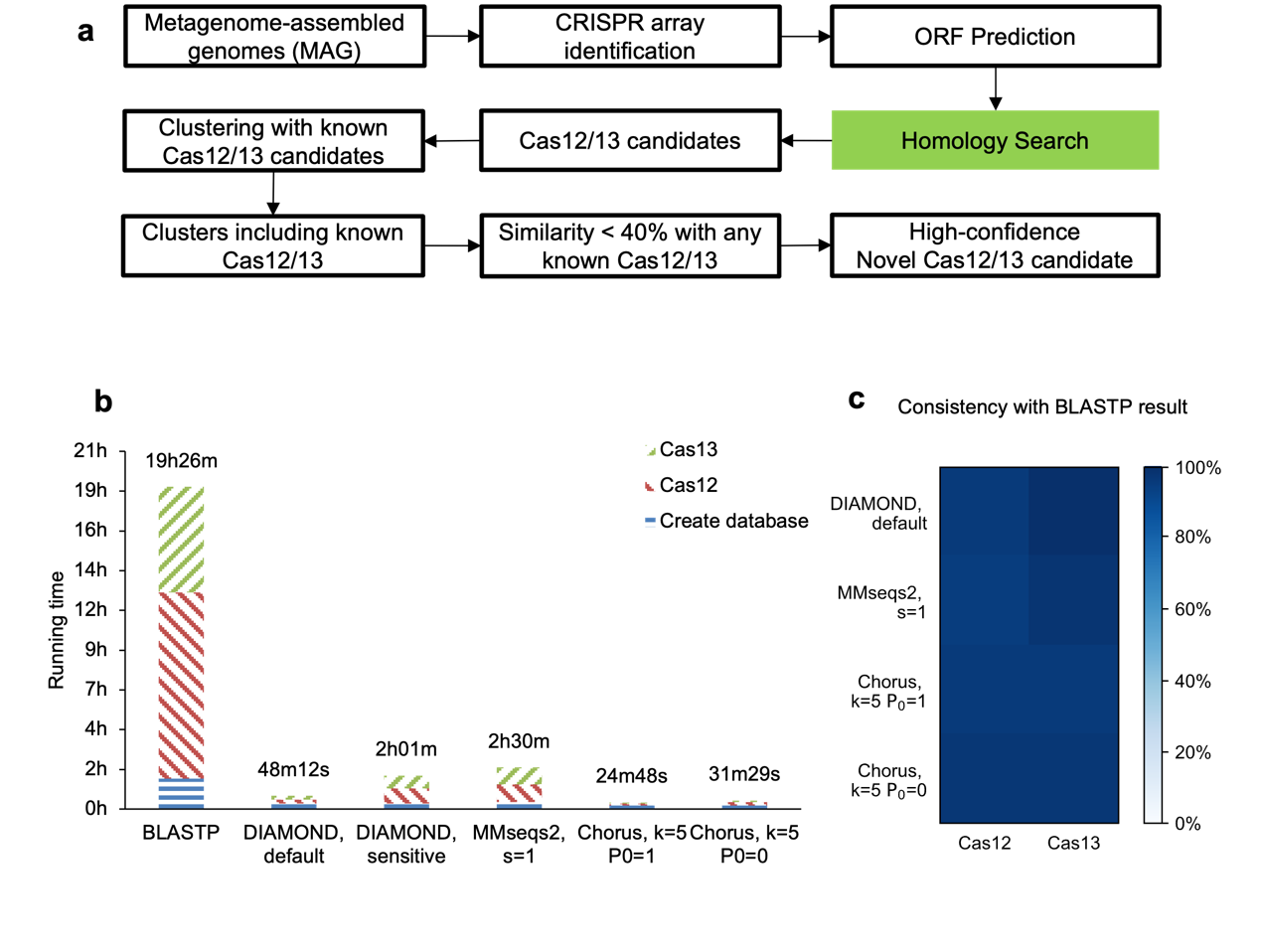


1. Pipeline of the novel Cas discovery. The homology search is using known Cas protein seqences to query against all ORFs found in genomes database.
2. Running time of querying known Cas12 and Cas13 against ORFs found in the genomes, including the time of create database.
3. The coverage of BLASTP query results of different methods.

## Design principle of Chorus

Chorus utilizes the CPU and GPU heterogeneous computing power (Figure 1). The key algorithmically innovation is the use of a more GPU-friendly voting algorithm instead of the ungapped extension in BLAST. And after preliminary filtering to obtain HSP (High Scoring Pair) candidates, banded local alignment is performed according to the approximate alignment positions. The above processes can form a pipeline between heterogeneous devices to achieve sufficient compression of CPU, GPU runtime and I/O time (SF1a).

We use the single-indexed search algorithm like blast. We first use CPU to concatenate and compress all database sequences end-to-end, then transfer them to GPU memory, keeping other descriptive information and their offsets separate. Using 5 bits instead of 8 to represent an amino acid letter reduces I/O time by 37.5% (SF1b), which is a substantial savings for large databases (over 100GB), especially since I/O is a big part of running time. For multiple query sequences, we build an index table for the consecutive k amino acids (k-mers), also transferred to GPU memory. Compared to database-indexed methods such as Diamond and Mmseqs, this strategy is more suitable for most query scenarios (up to thousands of query sequences against very large reference databases). Because the single-indexed method does not require a lot of memory and time to index the database.

We use the seed and vote algorithm instead of the widely used seed and extend. Existing GPU-accelerated blast methods (Vouzis and Sahinidis, 2011; Ye *et al.*, 2017) suffer from branch divergence in the parallel seed and extend stage. Because GPU threads execute instructions in SIMT mode, if some threads occur a hit (exact match of a seed), other threads that do not hit need to wait for them to complete the extend step before continuing to scan the next k-mer in database sequences. Our approach breaks the limit of scanning only one database sequence per thread and assigns each thread a database sequence of the exact same length. And we only record the number of hits (called votes) at a certain alignment position in a GPU hash table during the process of scanning the database, instead of doing the extend operation immediately, which minimizes the divergence among threads.

We use a banded local alignment algorithm to further compute and score HSP (high scoring pair) candidates whose number of hits (votes) exceeds a certain threshold. Since the approximate alignment position is already obtained in the seed and vote stage, we only need to perform the smith-waterman local alignment calculation near the diagonal line, thus minimizing the computation and memory usage to O(N), N is the length of the query sequence in the HSP. The current banded local alignment is implemented with CPU, and we will continue to develop GPU version in the future.

Finally, we combine these heterogeneous computations into a pipeline. We equally divide tasks into different streams, so that the most time-consuming I/O operations, GPU and CPU computations can overlap to minimize the total execution time. With proper parameter settings, these steps can be executed in about the same time to maximize the occupancy rate of all heterogeneous devices (SF1a).

## Preprocessing of large protein sequence database

Before the alignment task, Chorus needs to preprocess the large-scale protein sequence database, and each database only needs to be preprocessed once. The preprocessing is divided into two steps: compression and sequence merging. Database compression can effectively save memory and reduce data transmission costs, while sequence merging can relieve the thread divergence of GPU in subsequent calculations.

We first scan the database file in the original FASTA format and recodes each letter in all sequences, as shown in SF1b. Since the new coding range is 0 to 31, the storage space of each amino acid letter is reduced from 8 binary bits to 5, and the total memory space and transmission required for the database are actually reduced by 37.5%. Specifically, Chorus allocates a buffer of 20 bytes in the memory, and puts the converted amino acid letters into the buffer successively through bit operation. When the buffer is full, we write them to the disk as the pre-processed sequence file. For sequence description information in the FASTA file, Chorus puts it into another sequence description file separately and save it in text format, so that only simple sequence files need to be loaded into the GPU memory in the subsequent step, thus minimizing the memory storage and data transfer time.

In order to relieve the divergence caused by the different length of database sequences allocated to GPU threads in subsequent calculations, the existing GPU Protein Sequence Alignment Tools (Vouzis and Sahinidis, 2011; Ye *et al.*, 2017) sort all database sequences by length in advance in the preprocessing stage, so that when GPU scans the database, the length of sequences in the same warp will be closer as possible. But this still results in large load imbalance, cause the length of protein sequences can differ by factors of more than 100. In this paper, we break through the restriction that one single GPU thread should only process one database sequence. During the preprocessing, all database sequences are connected end to end, and a special symbol like ‘#’ is used as the interval between two sequences. In this way, each GPU thread allocates the database sequence with completely equal length so as to minimize thread divergence. In order to record the offset of each sequence in the sequence file and description file, we store these starting offsets in another file. Because the number of sequences in the database is far less than the number of amino acid letters, the size of the offset file is far less than that of the sequence file, and it is only loaded into the main memory at the final output stage. When accessing the information of a specific sequence in the output phase, since these offsets are ordered, and the starting offset of the alignment position in the database is known, by using binary search of the logarithmic complexity, we can quickly locate the corresponding sequence and description information.

## Seed index of the query sequences

In addition to the target protein sequence database, the query sequences are also required in FASTA format for each alignment task. This paper adopts the index method similar to BLAST(Altschul *et al.*, 1990), all the subsequences with the length of k in the query sequence are indexed, which is called k-mer. For the query sequences for a single alignment task is usually far less than the target database, thus the index table building time is negligible. However, more considerations are needed to facilitate the subsequent look-up operations.

Chorus uses an index table with a fixed number of rows and columns to store these indexes. Since there are 20 kinds of amino acids, and there are 20^k k-mers. The fixed size index table has 20^k rows, each row corresponds to a k-mer. Considering other letters and special characters that may appear in the sequence, Chorus expands the index table to 32^k rows, if the code of any letter in k-mer is 26 to 31, the corresponding row is a redundant area. In this way, it is convenient to base on the compressed database sequence to quickly address any k-mer in subsequent GPU calculations. Suppose the code of the i-th letter of any k-mer from left to right as $X_{i}$, then its number of rows in the index table can be quickly obtained through bitwise shift operation

$$\begin{aligned} X=\sum_{i=0}^{k-1} {32}^{i}X_{i}=\sum_{i=0}^{k-1} X_{i}\ll5i \end{aligned}$$

where $\ll$ is a bitwise left shift operator. For example, the seed length k=5 in SF1b, and the letter codes are $X_{0}X_{1}X_{2}X_{3}X_{4}$ respectively. The number of rows of a particular k-mer in the index table can be calculated by shifting $X_{0}X_{1}X_{2}X_{3}X_{4}$ to the left by 0, 5, 10, 15 and 20 bits respectively, and finally adding them.

Besides, each row of the index table has a fixed number of cells to store the different positions of the k-mer in the query sequence, that is, the number of columns in the index table. Since there are a total of 20^k different k-mers, most of k-mers will not appear in the query sequences, and it is less likely that the same k-mer will appear many times, therefore the number of columns is usually small, such as 3 or 4 columns. If the number of occurrences of the same k-mer exceeds the number of columns, the nearest and empty redundant rows can be used for overflow storage. Meanwhile the last column of the original row needs to be changed to the row offset of the overflow row. In addition, Chorus can make multiple query sequences share the same index table, so that they can be queried in parallel. Therefore, each position of the index table stores the id q of the query sequence and the first letter offset i of the current k-mer in the sequence.

It should be noted that due to the limitation of GPU memory in the subsequent Vote step, the number of query sequences is also limited by the total length of amino acid letters. If the input query sequences exceed the total length limit, they need to be divided into several groups in advance. And each group should establish an index table, so the total number of groups could be as small as possible on the premise of meeting the total length limit. For the sequence grouping strategy, Chorus uses a simple heuristic algorithm similar to the Multiple Knapsack Problem, that is, firstly sorts all sequences by length, and then uses the upper limit of the total length of the sequence as the "knapsack" size, and uses the best fit Greedy Strategy to group them.

Chorus uses a fixed size index table instead of Hash index table, its main advantage is to facilitate the subsequent GPU look-up and reduce the number of accesses to GPU global memory to improve performance. Since a k-mer may appear multiple times in the query sequences, the hash table or DFA table needs to store multiple indexes of the same k-mer in the form of linked list. However, when a k-mer hit occurs, all indexes of the current k-mer need to be accessed, which will increase the number of random memory access. The fixed size index table can store all indexes of the same k-mer near each other, which is more cache-friendly. However, the limitation is that the memory occupation increases exponentially with k, and the k-mer index is sparser when k is large, and the space utilization is low. Fortunately, the k of BLAST and Chorus is usually small, e.g., no more than 5, therefore the index table size is acceptable. If k continues to increase, we can use the Hash table as the index table instead. Besides, take the common methods of dealing with hash conflicts as an example, the Hash table often needs multiple accesses to confirm the look-up failure.

When scanning each k-mer of a database sequence, since the size of query sequences is usually much smaller than the database, there should be a relatively small number of k-mer hits with the query sequences, that is, most look-up to the index table should result in no hits. That will increase the average number of access the Hash table. And DFA tables also require multiple random memory accesses to confirm that no hits have occurred. Therefore, both the Hash table and the DFA table need to set up an additional fixed-size list to record whether each k-mer appears in the query sequences, so that when scanning the database, it can quickly determine whether the current k-mer has at least one hit. And the size of this extra list also increases exponentially with k.

There is an existing method (Huang *et al.*, 2021) that tries to put fixed-size index tables into GPU shared memory for faster access speed, but due to the limited space of shared memory, the length of query sequences must be strictly limited, such as less than 128 amino acids. And multiple sequences cannot be queried at the same time.

## GPU seed and vote algorithm

Chorus follows the typical GPU heterogeneous calculation process: transfer the data from main memory to GPU memory, GPU calculation, and transfer the calculation results from GPU memory to main memory. In the case of large-scale sequence databases, the phase of filtering candidate sequences from the database usually takes up most of the running time of the BLAST heuristic algorithm(Vouzis and Sahinidis, 2011). This paper uses the same idea as the existing GPU acceleration tools (Ye *et al.*, 2017; Vouzis and Sahinidis, 2011) to accelerate the Seed and Ungapped extension phases with GPU, while the difference is to replace the Ungapped extension with the Vote algorithm, which is more suitable for the GPU architecture.

Firstly, Chorus loads the pre-compressed database sequence file and the seed index table of the query sequences into the GPU memory, and allocates the database sequence of the same length to each GPU thread for scanning. For each k-mer in the compressed sequence accounts for 5*k consecutive binary bits, and k is generally not greater than 5, at most two consecutive integer data (2*4 bytes) can be read for a complete k-mer. For each GPU thread, each k-mer is read from the given scanning start point of database sequences, and then the corresponding row in the index table is directly accessed according to the code of the k-mer. If there is no index record in this row, it means that no hit occurred; otherwise, it indicates that there is at least one hit with the query sequence at the corresponding position of the database, and further calculation is required.

The existing alignment tools, including BLAST(Altschul *et al.*, 1990), multi-sequence acceleration tools DIAMOND (Buchfink *et al.*, 2021, 2014) and MMseqs2 (Steinegger and Söding, 2017) , and the existing GPU acceleration tools (Vouzis and Sahinidis, 2011; Ye *et al.*, 2017), they all perform the ungapped extension calculation immediately after the seed hit, that is, perform the bidirectional extension without considering the gaps from the hit point until the alignment score drops to a certain threshold. Chorus uses the Vote algorithm to replace the ungapped extension. Firstly, we calculate the offset p of the alignment position in the database, corresponding to the end of the query sequence (without considering gaps). The specific calculation is

$$\begin{aligned} p=L+x-y \end{aligned}$$

where x is the offset of the current k-mer in the database; y is the offset of k-mer in the query sequence which obtained by looking up the index table; L is the length of the current query sequence which stored in the GPU constant memory for fast access.

The p is calculated so that if two or more hits for the same query sequence give the same p, it means that these hits occur at the same alignment position, which is similar to the effect of the original ungapped extension. As shown in SF1c, two seeds hits belong to the same alignment position, and the calculated p value is 132. If the number of hits at a certain p value exceeds a certain threshold, this alignment location may have a good local alignment with high probability, therefore can be used as a candidate sequence for the next local alignment calculation step. This method of filtering candidate sequences is called Vote. Each hit at the alignment position p is considered as one "vote", and only those positions with the most votes can enter the subsequent calculation. The complete GPU scanning database and seed hit process are described in Algorithm 1.

| **Algorithm 1: GPU Seed** |
| --- |
| **INPUT:** Database sequences S, k-mer index table of query sequences T  **OUTPUT:** Voting hash table H  **BEGIN**  Initialize hash table H  For each GPU Threads parallel do:  s_begin, s_end are the start and end positions of sequences that allocated from S  For s_offset←s_begin to s_end do:  Read k-mer that started with aa at S[s_offset] from global memory  Calculate the code of k-mer: X←$\sum_{i=0}^{k-1} X_{i}\ll5i$  Look up index table for this k-mer: Hits←T(X)  If Hits not EMPTY:  For each (q,i) in Hits:  Calculate alignment position: p←len(q)+s_offset-i  **Vote(H[q],p)**  Return H  **END** |

In addition, due to the consideration of gaps in the alignment, Chorus aggregates the calculated p value through a fast bit operation in order to make the voting more focused. For example,

$$\begin{aligned} \mathrm{pack}_{8}\left( p \right)=(p&＆\sim7)|3 \end{aligned}$$

where "&" is the bitwise AND operator, "~" is the bitwise negation operator, and "|" is the bitwise OR operator. The effect of pack is to aggregate the values in the [x, x+8) range of p into the intermediate value x+3, where x is an integer multiple of 8 so that the hits of p in this range will be counted as the same alignment and the vote will be focused. We can also continue to expand the aggregate range of seed hits.

In order to store and count the "votes", Chorus allocates a hash table for each query sequence in GPU memory in advance, and the Hash table size is proportional to the length of the current query sequence and the total size of the database scanned. The key of the Hash table is defined as the alignment position p, and the corresponding value is the number of hit times at p, that is, the number of votes, as shown in SF1c. Each time a hit occurs and the p value is calculated, it is used as a key to access the Hash table. If the same key p is found, its corresponding value will be added by one; If it is not found, insert the current p value as the new key in the Hash table and initialize the value to 1.

In order to minimize each hash calculation and insertion, Chorus uses the Murmur3 hash algorithm, as well as linear detection method to deal with the hash conflict. That is, starting from the slot corresponding to the hash value and scanning in sequence until the searched key is found, or finding the empty slot, then inserting the current key into the empty slot.

In order to vote for multiple query sequences in parallel, each thread accesses the same index table containing multiple query sequences. Moreover, since each k-mer in database may hits in multiple query sequences in the index table, Chorus has to vote into Hash tables of different query sequences.

Besides, since the hash table is shared by GPU multi-threads and accessed in parallel, it is necessary to maintain the exclusive access to the slots in the table. Chorus firstly uses the atomic CAS (Compare-and-swap) operation to access the hash table slot and insert a new key, that is, to compare whether the key of the specified hash table slot is empty. If it is empty, the new key will be placed in this position. And the whole process is atomic. The CAS operation will return to the original key in this slot in any case, and we can check whether the insertion or search is successful based on this: if the return key is empty or equal to the current key p, it means that the insertion or search is successful, and we can continue to modify the value of this slot. Otherwise, it indicates that a hash conflict has occurred, and we need to use the linear detection method to access the next hash table slot and repeat the atomic CAS operation. When modifying a value, we also need to use the atomic operation Add, that is, add one to the value atomically to ensure the mutual exclusion of the adding votes operation. See Algorithm 2 for detailed GPU Vote description.

| **Algorithm 2: GPU Vote** |
| --- |
| **INPUT:** Voting hash table H, alignment position p  **OUTPUT:** Modified H  **BEGIN**  Aggregate neighbouring hits: p←pack(p)  Calculate Murmur hash value: first_slot←Hash(p) mod size(H)  slot←first_slot  do  slot←slot mod size(H)  Access the hash table slot: prev←atomicCAS(H[slot].key, EMPTY, p)  if (prev==EMPTY or prev==key):  Access success, and increase the number of votes: atomicAdd(H[slot].value)  break  else:  Access the next slot by linear probing method: slot←slot+1  While slot≠first_slot  Return H  **END** |

After the GPU scans all database sequences, the Hash table stores the alignment position p and the corresponding hit times. At this time, we use GPU to scan and filter the hash table, take out the corresponding p value of the hit times greater than a certain threshold, and input to the next phase of local alignment calculation as a candidate high scoring pair (HSP). Specifically, similar to scanning the database, each GPU thread is allocated a fixed size hash table interval for scanning. And all GPU threads save the adequate p value to another list in GPU memory allocated in advance. Besides, it is also necessary to maintain the exclusivity of the save operation. The method is that since the atomic Add operation will return the original value, it can be used to maintain a variable that record total number of HSPs, and shared by multiple threads. Finally, we only need to copy the filtered alignment position p back to the host main memory as the final calculation result of the GPU. See Algorithm 3 for detailed description of GPU filtering HSPs.

| **Algorithm 3: GPU Filter** |
| --- |
| **INPUT:** Voting hash table H, filter threshold P  **OUTPUT:** HSP list R, number of HSP n  **BEGIN**  Initialize HSP list R, n←0  For each GPU Threads parallel do:  h_begin, h_end is the start and end position of scanned region that allocated from H  For h_offset←h_begin to h_end do:  If H[h_offset].value ≥ P do:  r_offset←atomicAdd(n)  R[r_offset]←H[h_offset].key  Return R, n  **END** |

Compared with the Ungapped extension algorithm, the Vote algorithm simplifies the processing steps when the seed hit occurs by inserting the GPU Hash table in parallel. It reduces the possibility of thread divergence, and thus maximizes the GPU performance, and prevents the GPU thread from scanning and extending simultaneously.

In general, Chorus has borrowed the heuristic search algorithm idea of BLAST, and transplanted it to GPU, and redesigned the algorithm as well as implementation based on the hardware architecture of GPU. Chorus implements the parallel indexing of multiple query sequences and large-scale database scanning. In addition, the Chorus has also achieved the most simplification in data transmission between main memory and GPU memory.

## Parallel banded local alignment

Similar to other alignment tools, after obtaining the high scoring pairs (HSPs) from the database search, Chorus needs to calculate the local alignment score for these HSPs. Since this step of alignment needs to consider the gaps, the basic method is Smith-Waterman dynamic programming algorithm, and the corresponding steps in BLAST are Gapped extension and Traceback. Unlike the original Smith-Waterman algorithm which needs to calculate the complete m*n score matrix, BLAST (Altschul *et al.*, 1990) has obtained a local alignment result without considering the gaps in the previous Ungapped extension stage, which essentially represents a segment of diagonal cells in the score matrix，and it assumes that this segment must be on the optimal alignment path as shown in SF1e (middle). Based on this, BLAST can continue to expand diagonally and calculate the remaining undetermined elements in the matrix. SF1e is a schematic diagram of the calculation results of the score matrix in different local alignment methods. The darker the color is, the higher score this position gets, and the pure white is the area which does not need to be calculated. The red line obtained by Traceback is the optimal alignment path，which shows that the optimal path in different methods in this example is basically the same. In addition, BLAST Gapped extension (middle) can greatly reduce the amount of calculation by at least 50%, compared with full score matrix calculation(left), but they are both O(mn).

However, the result obtained by Chorus in the previous GPU calculation is only the alignment position p, which is also a diagonal line in the score matrix. It is known that there are many seed hits on (or near) this diagonal line, but no score calculation has been performed. Therefore, Chorus only needs to use the Smith-Waterman algorithm to calculate the score of the diagonal and a certain range of cells around it. As shown in SF1e (right), the specific calculation area is set as the cells whose distance from the diagonal is less than a certain constant w, and the rest of the unit scores are initialized to 0 and no longer calculated. This pruning strategy is also based on BLAST's assumption that "good similarity alignment is often accompanied by accurate local matching". This step is called banded local alignment for the calculation area takes on the shape of a band. Where w is a constant of computation width, the larger w is, the greater the maximum number of gaps allowed in the final matching result is, the more likely it is to approach the optimal solution obtained by Smith-Waterman algorithm for calculating the complete scoring matrix. But the cost is greater computation and memory footprint. On the contrary, if w is small, it may lose the optimal solution and obtain the suboptimal solution instead. But its computation and memory usage are lower. In addition, w should be larger than the pack aggregation range of seed matching, and at least all seed hits near the diagonal must be covered. The banded local alignment algorithm can reduce the square-time complexity of the original O(mn) to the linear complexity of O(min(m,n)), and the memory complexity can also be reduced to linear through the transformation of the dynamic programming score matrix. As the simple transformation diagram shown in SF1d, the blue cell is the diagonal line corresponding to the alignment position p, and the yellow arrow indicates the dependency of the state transition before and after the matrix transformation.

The existing GPU accelerated alignment tools, such as GPU-BLAST (Vouzis and Sahinidis, 2011) and H-BLAST (Ye *et al.*, 2017), have completely retained the Gapped extension in BLAST using CPU for calculation. Chorus also chooses CPU multi-thread local alignment. Since a large number of HSP local alignment tasks need to be executed in parallel, Chorus schedules these tasks by maintaining a fixed-size CPU thread pool and task queue. Each thread carries out a HSP alignment, and the remaining tasks enter the queue to wait for the execution of the previous thread tasks, which can avoid the extra scheduling overhead. When all tasks in the queue are executed, Chorus outputs all alignment results in the specified format.

## Asynchronous pipeline

In the algorithms described above, Chorus has implemented parallel scanning of large-scale database on GPU, parallel seeding of the query sequence and multi-thread parallel local alignment on CPU. In the calculation pipeline, each step must be executed serially due to the dependency, but the main time-consuming calculation steps are using different heterogeneous resources, such as database sequence transmission, GPU and CPU operations which can be executed asynchronously. However, the existing GPU acceleration tools (Vouzis and Sahinidis, 2011; Ye *et al.*, 2017) still maintain the serial flow of each step of BLAST, so the GPU has a lot of idle time after the end of the Seeding and Ungapped extension phases, as shown in SF1a. Chorus uses CPU-GPU asynchronous pipeline design, which enables the above core steps in the pipeline to be executed asynchronously and overlapped. The asynchronous pipeline can maximize the use of computing resources of heterogeneous computers, and significantly reduce the total running time.

Specifically, Chorus uses the CUDA stream technology to asynchronously execute the database sequence transmission and calculation. The sequential steps of the same CUDA stream cannot be executed at the same time due to the calculation dependency, and the same step of different streams also cannot be executed at the same time due to the limitation of computing resources. However, different steps of different streams can be executed asynchronously. Firstly, in the database preprocessing stage, Chorus divides the database sequence into several equal parts, each corresponding to the input of a stream, like 16 streams. More streams can theoretically increase the performance, but with diminishing marginal utility. For example, if the running time of the three steps is 1, the total running time of x streams is 1+1/x. When loading the database sequence from the disk, Chorus uses the memory mapping (mmap) technology to directly read the sequence file from the page cache in host memory without additional memory allocation and copying. And since this memory space cannot be automatically exchanged to virtual memory, it belongs to pinned memory, which can speed up the copy from main memory to GPU memory, and also support asynchronous transmission so that the data transmission of the current stream and the GPU calculation of the previous stream can be carried out simultaneously. To enable GPU and CPU computing to overlap, Chorus sets an event for the end of each CUDA stream. When the GPU computing task of the stream is completed and the calculated alignment position p is copied to the main memory, the event will be triggered. The CPU opens a thread for each stream to wait for the event, and then pushes all corresponding HSPs into the thread pool task queue for subsequent local alignment.

Ideally, the asynchronous pipeline design makes the total running time of the system become the maximum value from the sum of the running time of each step.

## Parameter settings

Although the GPU parallel algorithm is efficient enough, it is still limited by the size of GPU memory in the real large-scale protein sequence alignment task. For example, the current sequence database has exceeded the size of main memory and GPU memory, which can only be calculated by batch. In addition to the database sequence itself, the hash table storing the hit times of the Vote algorithm also needs a large amount of GPU memory. And the size of the hash table is related to the total number of hits that may occur among the current database batch and the query sequences. First, the total number of hits is theoretically proportional to the database size and the total length of the query sequences. Therefore, if the GPU memory is not enough to store the hash table, in addition to calculating the database in batches, we can also group the query sequences, create index tables and align them with the database respectively. However, it will increase the scanning times of database and cause performance degradation.

Besides, the length of the seed k also affects the total number of hits. The larger the k is, the smaller the number of hits that could theoretically occur, and the less GPU memory usage. As the distribution of seeds in the database cannot be assumed in advance, we use several random sequences to query the large sequence database NR as a test. The resulting distribution of the number of HSPs with different seed hits is shown in SF3. The horizontal axis represents the number of seed hits, and the vertical axis represents the number of HSPs with specified number of hits, and let the seed length k be 3, 4 or 5, respectively. It can be seen that when k increases by 1, the total number of hits and the GPU memory required for the hash table decrease by about one order of magnitude. Therefore, when the GPU memory is constant, a larger k can search and align larger databases as well as more query sequences in parallel with the improvement of the system's performance, but the cost is hurt sensitivity.

Furthermore, according to the experimental results in SF3, when the filtering threshold of votes P is small enough, the number of HSPs will also decrease by about one order of magnitude for each increase of P. Therefore, Chorus can set a dynamic filter threshold of votes for each query sequence, so that sequences with different lengths can get as same number of HSPs as possible. Specifically, when the length of the query sequence increases by one order of magnitude, the threshold also increased by one to offset the increase in the number of HSPs, namely

$$\begin{aligned} P=P_{0}+\log_{10} L \end{aligned}$$

Where L is the length of the query sequence, $P_{0}$ is a preconfigurable constant, such as 0 or 1. We can modify $P_{0}$ to set the overall threshold and sensitivity.

## References

Altschul,S.F. *et al.* (1990) Basic local alignment search tool. *J Mol Biol*, **215**, 403–410.

Buchfink,B. *et al.* (2014) Fast and sensitive protein alignment using DIAMOND. *Nat Methods*, **12**, 59–60.

Buchfink,B. *et al.* (2021) Sensitive protein alignments at tree-of-life scale using DIAMOND. *Nat Methods*, **18**, 366–368.

Huang,L.T. *et al.* (2021) A lightweight BLASTP and its implementation on CUDA GPUs. *Journal of Supercomputing*, **77**, 322–342.

Steinegger,M. and Söding,J. (2017) MMseqs2 enables sensitive protein sequence searching for the analysis of massive data sets. *Nat Biotechnol*, **35**, 1026–1028.

Vouzis,P.D. and Sahinidis,N. V. (2011) GPU-BLAST: Using graphics processors to accelerate protein sequence alignment. *Bioinformatics*, **27**, 182–188.

Ye,W. *et al.* (2017) H-BLAST: A fast protein sequence alignment toolkit on heterogeneous computers with GPUs. *Bioinformatics*, **33**, 1130–1138.
